# Supplementary material for: Smart city pilots, marketization processes, and substantive green innovation: A quasi-natural experiment from China
Source: PLoS One. 2023 Sep 27;18(9):e0286572. doi: 10.1371/journal.pone.0286572 (PMC10529645; doi:10.1371/journal.pone.0286572)
Supplement: Attachment — Submitted filename: Response to Reviewers.docx [file pone.0286572.s002.docx]

Response to Reviewers

Dear editor and reviewers,

Thank you for offering us an opportunity to improve the quality of our submitted manuscript (PONE-D-22-28078). We appreciated very much the reviewers constructive and insightful comments. In this revision, we have addressed all of these comments. We hope the revised manuscript has now met the publication standard of *PLOS ONE.*

Reviewer #1

Comment 1: I fear that this paper falls into the same trap that many similar quasi-natural experiment papers trigger: applying PSM-DID methodologies and robust techniques to data to find a pattern, without a detailed analysis of the components and dynamics of the underlying systems contributing to the academic community. The universe of time series functions is sufficiently large that something can usually be found, but this doesn´t help understand the underlying variables' interaction mechanism. This is a particularly nasty trap in correlation relationship study since very limited data about other components of its evolutionary behavior is available. Without a clear justification based on possible interaction mechanisms, which is absent, I don´t think it is valid to blindly apply techniques to investigate the task force on smart city pilots on the substantive green innovation of enterprises. A general problem with any empirical analysis (which often gets overlooked) is finding satisfactory methods to compensate for changes in the underlying quantities of the unit of measurement. In principle, this is something that some variables mentioned in the manuscript were supposedly designed to avoid, but in practice, this has not been the case so far. I strongly advise authors to firstly construct a novel theory which is verified by the following data analyses, otherwise data results are not enough to support your conclusions. I am convinced this manuscript studied a statistical correlation, not an economic one. I don´t find the empirical analysis compelling, or adding anything to the literature. Overall, there are a number of occasions in the paper where I think claims are being made without sufficient substantiation, and that the paper is also not robust to a suggestion of some degree of cherry picking.

Response: Thanks for the suggestions. The study we conducted was based primarily on the theory of government behavior and firm innovation, because China's smart city pilot is a typically integrated government action, there are strict requirements for companies in terms of digitalization and environmental protection. We have added the theoretical framework of government intervention behavior and firm innovation to the section 2.1. In the section 4.3, we have added potential mechanisms for smart city pilots to influence firm innovation: government subsidies to firms and digital transformation of firms.

Reviewer #2:

1.what's the relationship between the two or even three: Smart City Pilots, Marketization Processes, and Substantive Green Innovation. There is limited theorical analysis about this. How does Marketization Processes affect Substantive Green Innovation or vice versa.

Response: Thanks for the suggestions. The study we conducted was based primarily on the theory of government behavior and firm innovation. Smart city construction as a governmental action can affect firm innovation. In section 2.1 we have added the theoretical framework of government intervention behavior and firm innovation. In different cities, the degree of marketization varies, leading to different effects of smart city pilots on firm innovation. In Section 2.3, we analyze the theoretical basis of marketization as a moderator. We verify, in section 4.4 of the paper, the moderating effect of marketization through a moderating effect model. In fact, we want to explain that the government as the "visible hand" and the "invisible hand" of the market mechanism to cooperate with each other in order to build a conducive environment for the healthy development of enterprises.

2.How to set the hypothesis is not well founded. For example. "In reviewing the literature on smart cities, it is found that researchers generally agree that smart cities create a system of innovation". It's suggested to gather theorical model or analysis to support the hypothesis. The empirical analysis is only part of verifying the theory not a substitution of theory.

Response: Thanks for the suggestions. We have added the theoretical framework of government intervention behavior and firm innovation to the section 2.1. In the section 4.3, we have added potential mechanisms for smart city pilots to influence firm innovation: government subsidies to firms and digital transformation of firms.

3.the Moderating effect test is not sufficient without a theorical analysis.

Response: Thanks for the suggestions. We have added a theoretical analysis of the moderating effect to Section 2.3.

4.the endogenous problem must be addressed.

Response: Thanks for the suggestions. Regarding the endogeneity problem, we used a combination of PSM and DID. The PSM method can match each treatment group sample to a specific control group sample, making the quasi-natural experiment approximately random, thus well solving the endogeneity problem of selective samples.

Reviewer #3: The study found that smart city pilots drove substantial green innovation in businesses. Marketization process has moderating effect on the impact of smart city pilots on substantive green innovation of enterprises. Through further analysis, it is found that the marketization process has threshold effect in smart city pilots influencing the green substantive innovation of enterprises, and the effect of smart city driver influencing the substantive green innovation of enterprises increases significantly when regional marketization process reached a certain level. Lastly, based on the empirical results, relevant suggestions for optimizing smart city construction are proposed to lay the foundation for further exerting the good effect of smart cities. The topic is interesting for journal readers. The suggestion is to improve the discussion of the results in terms of policy implications. Moreover, the text should be English proofread because some sentences are not clear.

Response: Thanks for the suggestions. We've added a part for government-specific recommendations in section 6.
