# Supplementary material for: Smart city pilots, marketization processes, and substantive green innovation: A quasi-natural experiment from China
Source: PLoS One. 2023 Sep 27;18(9):e0286572. doi: 10.1371/journal.pone.0286572 (PMC10529645; doi:10.1371/journal.pone.0286572)
Supplement: Attachment — Submitted filename: Reviewer.doc [file pone.0286572.s003.doc]

By reading the author's paper, I found several more serious problems.

1. the writing structure of the Chinese paper. The authors should read more English papers and understand the English paper writing structure instead of directly translating Chinese papers into English. As an example, the article lacks a follow-up research arrangement in the introduction section.

2. the references are too old. Most of the papers are before 2019, and many are even 2009. hopefully, the authors can recognize the importance of the literature review. In addition, I hope you can compare with the existing literature in the literature review to highlight the strengths of your research.

3. the authors should follow the structure of research objectives-methods-results-conclusion in the writing of the abstract.

4. The introduction is really poorly written. After reading the introduction I didn't understand the background of your study and the progress of the current study. The introduction is a very important part of the article. However, the author has neglected to do so. In the introduction, the author should give an overview of the background, significance and development of the study. Point out the important advances in current research and the gaps in current research. Based on this, introduce the purpose of their research. Finally highlight their research contribution and summarize the overall paper's research.

5. Hypotheses 2 and 3 are formulated too arbitrarily and without any theoretical support at all.

6. The author's methodological design is too arbitrary and I hope it can be redesigned.

7. I did not find fig1 and fig2, however, the authors cited two figures.

8. the authors' conclusion is poorly written. the conclusion section briefly summarizes the study of the paper and then points out the shortcomings of the study.

9. The authors' study lacks a discussion section.

The author's current research is still at a poor level. Then the topic of the author's research is still relatively attractive. I hope the author can revise the article seriously.
