# Supplementary material for: Smart city pilots, marketization processes, and substantive green innovation: A quasi-natural experiment from China
Source: PLoS One. 2023 Sep 27;18(9):e0286572. doi: 10.1371/journal.pone.0286572 (PMC10529645; doi:10.1371/journal.pone.0286572)
Supplement: Attachment — Submitted filename: Response to Reviewers.docx [file pone.0286572.s004.docx]

Response to Reviewers

Dear editor and reviewers,

Thank you for offering us an opportunity to improve the quality of our submitted manuscript (PONE-D-22-28078). We appreciated very much the reviewers constructive and insightful comments. In this revision, we have addressed all these comments. We hope the revised manuscript has now met the publication standard of *PLOS ONE.*

Reviewer #4:

1. Although the introduction does a good job as explaining the importance of smart city construction, it does not clearly pose the research question, why did the paper aim to find out the intrinsic relationship between the construction of smart city and substantive green innovation of enterprises, and why did the paper take marketization into consideration? Besides, the three contributions need more work to be highlighted and clarified based on specific gaps, and suggesting that contributions can be elaborated in the section of Introduction.

Response: Thanks for the suggestions. We have added lines 29–74 in the revised version, detailing the goal of the article: to explore the relationship between smart city construction and enterprise green innovation. Lines 68–74 explain why marketization is being added to examine the connection between smart city construction and corporate innovation. Lines 75–86 provides a detailed explanation of the gaps and the contributions of this article.

2. Although the revised paper has added the theoretical framework in the section 2.1, it still failed to well examine the possible relationship between smart cities and green innovation. Specifically, I agree with the idea that smart city pilot is a kind of government behaviors, while not each government behavior will affect green innovation, thus suggesting that its theoretical logic should be explained much more in detail in the sections of Theoretical framework and Hypothesis development.
Response: Thanks for the suggestions. In the revised version, we explained much more in detail in Section 2 about theoretical logic and hypothesis development. We explained in more detail on lines 92–144 how the government's smart city construction affects the innovative behavior of enterprises.

3. Robustness tests were mainly according to the main effect of smart cities on green innovation, so it is better that the section 5 could be removed to behind of section 4.2. Additionally, besides PSM, other methods could be suggested to test endogeneity comprehensively.
Response: Thanks for the suggestions. In the revised version, the section on robustness testing was moved behind Section 4.2, according to your suggestions, and the IV method for the robustness test was added. Therefore, we use the PSM method in combination with the DID and IV methods to comprehensively solve endogeneity problems. The detailed steps and results are shown on lines 207–257.

4. The revised version has added the underlying plausible mechanism for smart city pilots to influence firm innovation: government subsidies to firms and digital transformation of firms in the section 4.3, while there is no section 2.4 in this paper as mentioned on page 14, so the explanations of mediating variables or potential mechanisms were missing.

Response: Thanks for the suggestions. This is due to a change in the structure of the paper, resulting in a change in chapter position, which was our mistake. Regarding the explanations of mediating variables or potential mechanisms on lines 107–140.
5.This paper lacks a deep discussion to engage the literature, suggesting you could compare your study with the relevant literature in details, and show why your findings matter theoretically. Additionally, some limitations or future directions could be proposed.

Response: Thanks for the suggestions. In the new revised version, discussion is displayed on lines 331–351.We have added the "Limitations and future research" section on lines 376–384.

Reviewer #5:

1. the writing structure of the Chinese paper. The authors should read more English papers and understand the English paper writing structure instead of directly translating Chinese papers into English. As an example, the article lacks a follow-up research arrangement in the introduction section.

Response: Thanks for the suggestions. In the new revised version, we have redesigned according to the writing conventions of English papers. Specifically, first we added a follow-up research arrangement in the introduction section. Secondly, the specific chapters of the paper include the following parts: Section 1 is an introduction; Section 2 is a literature review and hypothesis development; and Section 3 presents the research methodology, data, and variable definitions; Section 4 contains the empirical results; Section 5 is the discussion; Section 6 outlines the policy implications; and Section 7 is about the limitations and future research.

2. the references are too old. Most of the papers are before 2019, and many are even 2009. hopefully, the authors can recognize the importance of the literature review. In addition, I hope you can compare with the existing literature in the literature review to highlight the strengths of your research.

Response: Thanks for the suggestions. In the new revised version, we have redesigned the literature review section. We have deleted some old literature and added important new literature, as reflected in Sections 1 and 2 of the paper.

3. the authors should follow the structure of research objectives-methods-results-conclusion in the writing of the abstract.

Response: Thanks for the suggestions. In the new revised version, we have considered your and other reviewers' opinions and rewritten the abstract section according to Background, Objective (s), Methods, Results, Conclusions, Policy Recommendations. Specifically, on lines 10-22.

4. The introduction is really poorly written. After reading the introduction I didn't understand the background of your study and the progress of the current study. The introduction is a very important part of the article. However, the author has neglected to do so. In the introduction, the author should give an overview of the background, significance and development of the study. Point out the important advances in current research and the gaps in current research. Based on this, introduce the purpose of their research. Finally highlight their research contribution and summarize the overall paper's research.

Response: Thanks for the suggestions. In the new revised version, we have rewritten the introduction. Specifically, we give an overview of the background, significance, and development of the study, point out the important advantages of current research and the gaps in current research, highlight research contributions, and summarize the overall paper's research. Specifically reflected in lines 26-90.

5. Hypotheses 2 and 3 are formulated too arbitrarily and without any theoretical support at all.

Response: Thanks for the suggestions. In the new revised version, we have rewritten the hypothesis section. Specifically, we provided a more detailed explanation of the theoretical logic behind the hypothesis. Specifically reflected in lines 92–140.

6. The author's methodological design is too arbitrary and I hope it can be redesigned.

Response: Thanks for the suggestions. In the new revised version, we use the PSM method in combination with the DID and IV methods to comprehensively verify the conclusions of the paper.

7. I did not find fig1 and fig2, however, the authors cited two figures.

Response: Thanks for the suggestions. In the new revised version, figs. 1 and 2 are behind page 28. It should be noted that the PDF file formed in the editing system contains two versions of the paper: one is the normal revised manuscript and the other is the revised manuscript with tracked changes. The former has figures, and the latter does not.

8. the authors' conclusion is poorly written. the conclusion section briefly summarizes the study of the paper and then points out the shortcomings of the study.

Response: Thanks for the suggestions. In the new revised version, we have redesigned the structure of the paper, and the last three parts of the paper include discussion, policy implications, limitations, and future research, as detailed in lines 330–384.

9. The authors' study lacks a discussion section.

Response: Thanks for the suggestions. In the new revised version, we have redesigned the structure of the paper, and the last three parts of the paper include discussion, policy implications, limitations, and future research, as detailed in lines 330–384.

Reviewer #6:

1. Abstract: The abstract should be written in the following context: Background, objective(s), methods, results, conclusions, policy recommendations.

Thanks for the suggestions. In the new revised version, we have considered your and other reviewers' opinions and rewritten the abstract section according to Background, Objective (s), Methods, Results, Conclusions, Policy Recommendations. Specifically, on lines 10-22.

2.Introduction: The introduction lacks study background. What is the novelty?

Thanks for the suggestions. In the new revised version, we have rewritten the introduction. Specifically, we give an overview of the background, significance, and development of the study, point out the important advantages of current research and the gaps in current research, highlight research contributions, and summarize the overall paper's research. Specifically reflected in lines 26-90.

3.How is your study different from other studies? Please explain in detail.

Thanks for the suggestions. Firstly, we examine the impact of smart city construction on microenterprises; previous research has mostly focused on the macro level. Secondly, we have added a very important element of marketization to the construction of smart cities and enterprise innovation behavior, as detailed in lines 56–86.

4.Many important studies related to sustainable development have been ignored. For instance, consult the following studies and improve your study:
<https://doi.org/10.1007/s11356-023-25662-w>
<https://doi.org/10.1080/1331677X.2022.2159849>
<https://doi.org/10.1007/s11356-022-24899-1>
<https://doi.org/10.1007/s11356-022-24387-6>
<https://doi.org/10.1007/s11356-022-24286-w>

Response: Thanks for the suggestions. In the new revised version, we have redesigned the literature review section. We have deleted some old literature and added important new literature, as reflected in Sections 1 and 2 of the paper.

5.How you can compare your results with other studies of same geographical regions. It is pivotal to tie your results with other relevant literature.

Response: Thanks for the suggestions. In the section of discussion, we tied our results with other relevant literature, as shown in lines 331-351.

6.Discussion: The study discussion is very generic and do not stem from study findings. It is suggested to rearrange discussion based on study findings.

Response: Thanks for the suggestions. In the new revised version, we have rearranged the discussion based on study findings as detailed in lines 331–351.

7.Conclusions: Conclusions are very weak and miss several important dimensions. To strengthen the contents and quality of the study, conclusions must be revised for more clarity and for the ease of normal readers.

Response: Thanks for the suggestions. In the new revised version, we have redesigned the structure of the paper, and the last three parts of the paper include discussion, policy implications, limitations, and future research, as detailed in lines 330–384. It is more clarity and for the ease of normal readers.

8.Policy recommendations: Specific policy recommendations should be put forward according to the target sample. General policies are of no use in scholarly articles.

Response: Thanks for the suggestions. In the new revised version, we have redesigned the section on policy implications, as detailed in lines 353–374. It is more specific and detailed.

9.Study limitations should be provided along with future research directions for prospective scholars interested in the similar works.

Response: Thanks for the suggestions. In the new revised version, we have added the section on limitations and future research, as detailed in lines 375–384.

10.The authors have used several old references to support their arguments. We are in 2023 and you are using such old references. In order to nurture the importance of study, references should be updated using recent and relevant studies.

Response: Thanks for the suggestions. In the new revised version, we have redesigned the literature review section. We have deleted some old literature and added important new literature, as reflected in Sections 1 and 2 of the paper.

11.There is an intermingle of capital and small letters. Please avoid this practice in scientific writing.
Response: Thanks for the suggestions. In the new revised version, we have checked and corrected the grammar and letter capitalization issues throughout the entire paper.

12. Finally, the manuscript can be benefited if the authors thoroughly proofread it in terms of English language mistakes and syntax structure.

Response: Thanks for the suggestions. In the new revised version, we have thoroughly proofread it in terms of English language mistakes and syntax structure.
