# Supplementary material for: Smart city pilots, marketization processes, and substantive green innovation: A quasi-natural experiment from China
Source: PLoS One. 2023 Sep 27;18(9):e0286572. doi: 10.1371/journal.pone.0286572 (PMC10529645; doi:10.1371/journal.pone.0286572)
Supplement: Attachment — Submitted filename: Response to Reviewers.docx [file pone.0286572.s005.docx]

Response to Reviewers

Dear editor and reviewers,

Thank you for offering us an opportunity to improve the quality of our submitted manuscript (PONE-D-22-28078). We appreciated very much the reviewers constructive and insightful comments. In this revision, we have addressed all these comments. We hope the revised manuscript has now met the publication standard of *PLOS ONE.*

Journal Requirements:

Please review your reference list to ensure that it is complete and correct. If you have cited papers that have been retracted, please include the rationale for doing so in the manuscript text, or remove these references and replace them with relevant current references. Any changes to the reference list should be mentioned in the rebuttal letter that accompanies your revised manuscript. If you need to cite a retracted article, indicate the article’s retracted status in the References list and also include a citation and full reference for the retraction notice.

Thanks for the suggestions. We checked all references one by one and corrected them as follows:

9.	Han Y. Smart Growth, Smart City. In: 2017 International Conference on Machinery, Electronics and Control Simulation (mecs 2017). Paris: Atlantis Press; 2017. p. 580–3.

22.	Shelton T, Zook M, Wiig A. The ‘actually existing smart city.’ Cambridge Journal of Regions. 2015; 8:13-25. https://doi.org/10.1093/cjres/rsu026

28.	Nam T, Pardo TA. Conceptualizing smart city with dimensions of technology, people, and institutions. In: the 12th Annual International Digital Government Research Conference on Digital Government Innovation in Challenging Times. Maryland: ACM Press; 2011. p. 282-91. https://doi.org/10.1145/2037556.2037602

32.	Sun B, Ruan A, Peng B, Liu S. Pay disparities within top management teams, marketization and firms’ innovation: evidence from China. Journal of the Asia Pacific Economy. 2022;715–35. https://doi.org/10.1080/13547860.2020.1865248

Additional Editor Comments:
The section structure of this manuscript does not make sense. Please redesign the section structure, a conclusion section should be located after the discussion section. Perhaps you should reorganize the layout of the section structure for Conclusions, Policy implications and Limitations and future research.

Response: Thanks for the suggestions. The conclusion section has been located after the discussion section in the revised version. Meanwhile, we reintroduced the structure of the article in the introduction section. Specifically reflected in lines 87–90 and 352-361.

Reviewer #1: All comments have been addressed

Reviewer #3: All comments have been addressed

Reviewer #4: This review concerns the revised manuscript titled “Smart City Pilots, Marketization Processes, and Substantive Green Innovation: A Quasi-Natural Experiment from China” (PONE-D-22-28078R2). The authors have made more efforts to improve the writing quality and revise the concerns mentioned by the reviewers. In this revised manuscript, I feel that it can be accepted for publication in this journal.

Reviewer #5: All comments have been addressed

Reviewer #6: All comments have been addressed

We sincerely thank the reviewers and editor for their valuable comments, which are of constructive significance to the improvement of this paper.
